# Fat Emulsion Intragastric Stability and Droplet Size Modulate Gastrointestinal Responses and Subsequent Food Intake in Young Adults^1^,^2^,^3^,^4^

**DOI:** 10.3945/jn.114.204339

**Published:** 2015-04-29

**Authors:** Mahamoud O Hussein, Caroline L Hoad, Jeff Wright, Gulzar Singh, Mary C Stephenson, Eleanor F Cox, Elisa Placidi, Susan E Pritchard, Carolyn Costigan, Henelyta Ribeiro, Elisabetta Ciampi, Asish Nandi, Nick Hedges, Paul Sanderson, Harry PF Peters, Pip Rayment, Robin C Spiller, Penny A Gowland, Luca Marciani

**Affiliations:** 5 Sir Peter Mansfield Magnetic Resonance Centre, School of Physics and Astronomy, and; 6 Gastrointestinal Surgery, University of Nottingham, Nottingham, United Kingdom;; 7 Nottingham Digestive Diseases Centre and National Institute for Health Research Biomedical Research Unit, Nottingham University Hospitals, University of Nottingham, Nottingham, United Kingdom;; 8 Unilever Discover, Sharnbrook, United Kingdom; and; 9 Unilever Research and Development, Vlaardingen, The Netherlands

**Keywords:** magnetic resonance imaging, physical form of food, lipid, food intake, stomach, small bowel

## Abstract

**Background:** Intragastric creaming and droplet size of fat emulsions may affect intragastric behavior and gastrointestinal and satiety responses.

**Objectives:** We tested the hypotheses that gastrointestinal physiologic responses and satiety will be increased by an increase in intragastric stability and by a decrease in fat droplet size of a fat emulsion.

**Methods:** This was a double-blind, randomized crossover study in 11 healthy persons [8 men and 3 women, aged 24 ± 1 y; body mass index (in kg/m^2^): 24.4 ± 0.9] who consumed meals containing 300-g 20% oil and water emulsion (2220 kJ) with *1*) larger, 6-μm mean droplet size (Coarse treatment) expected to cream in the stomach; *2*) larger, 6-μm mean droplet size with 0.5% locust bean gum (LBG; Coarse+LBG treatment) to prevent creaming; or *3*) smaller, 0.4-μm mean droplet size with LBG (Fine+LBG treatment). The participants were imaged hourly by using MRI and food intake was assessed by using a meal that participants consumed ad libitum.

**Results:** The Coarse+LBG treatment (preventing creaming in the stomach) slowed gastric emptying, resulting in 12% higher gastric volume over time (*P* < 0.001), increased small bowel water content (SBWC) by 11% (*P* < 0.01), slowed appearance of the ^13^C label in the breath by 17% (*P* < 0.01), and reduced food intake by 9% (*P* < 0.05) compared with the Coarse treatment. The Fine+LBG treatment (smaller droplet size) slowed gastric emptying, resulting in 18% higher gastric volume (*P* < 0.001), increased SBWC content by 15% (*P* < 0.01), and significantly reduced food intake by 11% (*P* < 0.05, equivalent to an average of 411 kJ less energy consumed) compared with the Coarse+LBG treatment. These high-fat meals stimulated substantial increases in SBWC, which increased to a peak at 4 h at 568 mL (range: 150–854 mL;* P* < 0.01) for the Fine+LBG treatment.

**Conclusion:** Manipulating intragastric stability and fat emulsion droplet size can influence human gastrointestinal physiology and food intake.

## Introduction

When fat is emptied from the stomach into the small bowel, long-chain FAs, the products of intraintestinal digestion, trigger active responses from the duodenum with impacts throughout the gastrointestinal tract (1), influencing hunger and food intake (2). The mode of delivery of the fat into the small intestine and its digestion are strongly influenced by the key variables of intragastric spatial distribution and the surface area of fat (3).

Early radiolabel studies provided evidence that the isolated lipid component of a test meal empties much more slowly than the aqueous component of the meal (4–6), whereas lipid integrated within the food matrix empties with the meal (7). MRI studies have demonstrated the importance of fat layering by showing how posture alters gastric emptying of a multiphase aqueous and oil meal (8). They also showed that fat emulsions that are stable in the acidic gastric environment empty more slowly from the stomach than emulsions that break and layer, possibly by increasing cholecystokinin (CCK)^10^ hormonal response and hence the sense of satiety (9, 10).

There is evidence that fat emulsion droplet size is also a key physicochemical variable affecting gastrointestinal function and satiety (3), both from early studies in animal models (11) and humans (12) whereby increasing the size of droplets in fat emulsions delivered directly to the small intestine reduced gastric antral pressure waves and attenuated plasma CCK and peptide YY (PYY) responses. Hunger and energy intake were reduced (12). In another study, emulsified fat, again delivered intraduodenally, increased CCK, pancreatic polypeptide (PP), and gallbladder contraction compared with unemulsified fat (13). In another key study, coarse (10-µm) and fine (0.7-µm) fat emulsions were delivered intragastrically and gastric and duodenal aspirates were taken at intervals to measure fat droplet size and lipase activity (14). Gastroduodenal lipolysis was higher for the fine emulsion (14). The “droplet size effect” can be explained by the fact that, for a given mass of fat, a smaller droplet size offers a larger lipid surface area (14). The increased surface area will, in turn, allow a higher number of lipase molecules to bind at the oil-water interface because lipase is generally present in excess (15, 16). The infusion of small amounts (6 g) of a fine and a coarse fat emulsion showed that the effect of droplet size is intestinal site specific (17).

However, intubation is potentially noxious and alters gastric behavior, so it is important to investigate the link between manipulation of the microstructure of fat emulsions, intragastric behavior, and gastrointestinal and satiety responses noninvasively by using physiologic oral ingestion of the test meals. We did this using MRI with the aim to test the hypotheses that gastrointestinal physiologic responses and satiety will be increased by the following: *1*) an increase in intragastric stability of a fat emulsion with a given fat droplet size and *2*) a decrease in fat droplet size of a fat emulsion with increased intragastric stability. In this context, intragastric stability means the prevention of lipid layering/creaming and coalescence in the stomach, which is achieved by adding a food thickener, locust bean gum (LBG), to the emulsion meals.

## Methods

### 

#### Test meals.

The 3 fat emulsion meals used here were isoenergetic (2220 kJ) and isovolumetric and differed only in their intragastric stability and droplet size. The 3 treatments were as follows: *1*) 300-g oil and water emulsion of 20% sunflower oil (containing 100 mg ^13^C-labeled octanoic acid) and 1% Tween 20, with 6 μm surface-weighted mean droplet size (Coarse); *2*) 300-g oil and water emulsion of 20% sunflower oil (containing 100 mg ^13^C-labeled octanoic acid), 1% Tween 20, 0.5% LBG, with 6-μm surface-weighted mean droplet size (Coarse+LBG); and *3*) 300-g oil and water emulsion of 20% sunflower oil (containing 100 mg ^13^C-labeled octanoic acid), 1% Tween 20, 0.5% LBG, with 0.4-μm surface-weighted mean droplet size (Fine+LBG).

**FIGURE 1:**
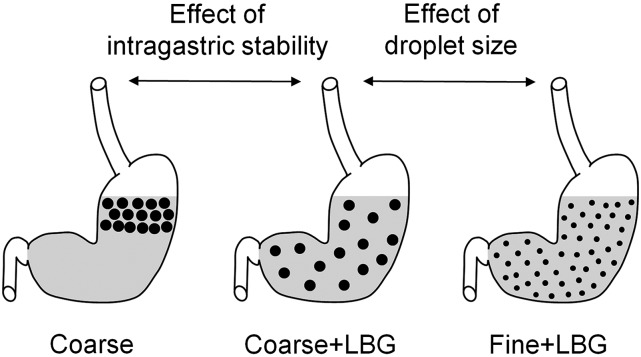
Schematic diagram of the predicted behavior in the stomach of the 3 meals containing 20% sunflower oil in water emulsions: Coarse, Coarse+LBG, and Fine+LBG. The black dots represent the fat droplets, which are small for the Fine emulsion and large for the Coarse and Coarse+LBG emulsions. The arrows indicate the predicted different appearance of the treatments in the stomach when changing intragastric stability and droplet size of the emulsions. Coarse, 20% oil and water emulsion with 6-μm mean droplet size; Coarse+LBG, 20% oil and water emulsion with 6-μm mean droplet size and 0.5% locust bean gum; Fine+LBG, 20% oil and water emulsion with 0.4-μm mean droplet size and 0.5% locust bean gum; LBG, locust bean gum.

Their predicted behavior within the gastric lumen is schematically represented in **Figure 1**. Flavoring was added to improve palatability. The ingredients and details of the preparation of the fat emulsions meals are given in the **Supplemental Information**. Each fat emulsion was prepared individually and had a unique identifier number assigned, and its preparation was recorded in a specially designed Standard Operation Procedures checklist. The droplet size was assessed for each sample as soon as it had been prepared, as described in Supplemental Information. The surface area–weighted mean D[3,2] was used as a measure of droplet distribution because it better reflects the surface area than the volume. Examples of droplet size distributions and of rheological profiles of the emulsions are given in **Supplemental Figures 1** and **2**.

#### Participants and study design.

Eleven healthy volunteers were recruited from the general university campus population and participated in this single-center, double-blind, randomized crossover study. There were 8 male and 3 female participants [aged 24 ± 1 y; BMI (in kg/m^2^): 24.4 ± 0.9] who were suitable for MRI scanning (i.e., no metal implants). They were asked to attend on 3 mornings 1 wk apart. They were asked to fast from 2000 h the previous evening and to avoid alcohol and any medication that could affect gastrointestinal function for 24 h and to avoid caffeine and strenuous exercise for 18 h before the experiment.

On each occasion, the abdomen was imaged at baseline (t = −1 h) to ensure the participants had an empty stomach and to acquire fasting baseline measurements of small bowel water content (SBWC). After this, the participants were taken out of the scanner and at time t = −15 min they were given 300 g of one of the above emulsions meals, which was chosen following a Latin square randomization schedule to avoid order effects. All meals were provided to the volunteer at 37°C to avoid rapid changes in relaxation times due to changes in temperature. Volunteers were positioned on the scanner table carefully, keeping them tilted slightly on their right side with the use of padding to encourage a normal “upright” gastric emptying profile, because previous research has shown that lying with the left side down (left lateral position) allows some fat from an oily meal to be delivered to the duodenum early, resulting in delayed gastric emptying of the entire meal (8). The participants were scanned every hour for ∼15 min for 5 h after the start of meal ingestion. After each set of scans, satiety visual analog scale (VAS) scores were collected. At regular intervals (at baseline and, after feeding, every 15 min for 2 h and subsequently every 30 min for up to 5 h), the subjects were asked to blow into ^13^C breath-test bags, which were stored for analysis, and serial blood samples were taken to measure CCK plasma concentrations. At the end of the MRI scans (t = 5 h), ad libitum food intake was assessed.

The volunteers were blinded to which meal they had been given. Every data file name was then changed according to a blind code by one person of the investigators’ team who was not involved in the study so that the operators were blinded to which data set they were processing, making the study a double-blind study. The code was broken only after all data had been processed and had undergone a blind data review.

The primary outcome was gastric emptying. Secondary outcomes were SBWC, magnetic resonance spectroscopy (MRS) of the stomach contents, ^13^C breath test, and assessment of satiety and food intake. This protocol was approved by the University of Nottingham Medical School Research Ethics Committee, and all participants gave informed written consent before experiments.

#### MRI and MRS.

MRI scanning was carried out on a 1.5-T Philips Achieva MRI scanner (Philips Healthcare) by using a 16-element parallel-imaging coil wrapped around the abdomen. A range of abdominal scans were acquired in ∼15 min for each time point. A balanced gradient echo (balanced turbo field echo) sequence was used to quantify gastric volumes. This imaging sequence yields good signal from the fat emulsions, which appear brighter than other abdominal organs, such as liver and spleen. Forty contiguous 7-mm-thick transverse slices were acquired in 2 separate expiration breath-holds lasting 13 s each, covering the whole abdomen [flip angle = 80°, repetition time (TR) = 2.8 ms, echo time (TE) = 1.4 ms, field of view = 400 mm, in-plane resolution = 1.56 × 1.56 mm^2^, acceleration factor = 2.0].

Proton spectroscopy was acquired by using stimulated-echo acquisition mode (90° × 90° × 90°), TR = 4 s, TE = 9 ms, 2 dummy scans, voxel size = 25 × 25 × 25 mm^3^, spectral bandwidth = 1000 Hz, 512 samples, and 4 repeats acquired in 24 s (18). This was acquired separately for 2 voxels, one in the upper (top) and one in the lower (bottom) parts of the gastric lumen corresponding to different components of the gastric contents due to layering in the supine position as identified on the balanced turbo field echo images.

SBWC was imaged as previously described and validated (19, 20) by using a coronal, single-shot, fast spin echo sequence. This sequence yields high-intensity signals from areas with freely mobile fluid and little signal from other body tissues. Twenty-four coronal images were acquired with TE = 320 ms, TR = 8000 ms, refocusing flip angle = 150°, fat saturation (using the spectral presaturation with inversion recovery pulse), in a single 24-s expiration breath-hold, with reconstructed in-plane resolution of 0.78 × 0.78 mm^2^ and slice thickness of 7 mm. At each time point, the positioning of the participant on the scanner bed, set-up, scout imaging, and data collection took ∼15 min, after which the participants were taken out of the scanner and kept sitting upright in a quiet room next to the scanner.

#### Satiety and food intake.

Participants were asked to fill out 100-mm VASs (21, 22) on paper each time they came out of the scanner. They were asked to rate their sense of fullness (anchored from “not full” to “extremely full”), hunger (anchored from “not hungry” to “extremely hungry”), and prospective food consumption (anchored from “nothing” to “an enormous meal”) (9, 10).

Food intake was measured at 5 h after consumption of the test meal by providing an ad libitum meal consisting of a single dish in very large quantities. Food intake from this meal was calculated from the difference in the weight of food offered and that remaining. The ad libitum meal was a standardized tomato-and-mozzarella pasta bake, purchased ready-made from a supermarket (Sainsbury’s) and heated by using a microwave oven. The macronutrient composition per 100 g of cooked product was as follows: 507 kJ energy, 5 g protein, 17.9 g carbohydrate, 3.2 g fat, 2.1 g fiber, and 0.45 g salt.

#### Breath samples.

The ^13^C octanoic acid substrate that was added to the fat emulsion meals would have been rapidly absorbed in the small bowel and metabolized to ^13^CO_2_ and finally excreted in the breath. As such, assuming the label was entirely associated with the emulsified fat, this will represent a marker of small bowel availability and absorption of fat in the meal. Breath samples were collected in breath bags and later analyzed by using an infrared isotope analyzer (IRIS; Wagner Analysen Technik). The ^13^C-labeled octanoic acid substrate ingested with the fat emulsion meals was metabolized and excreted in the breath. The isotope analyzer determined the 2 isotopes ^13^CO_2_ and ^12^CO_2_ in the breath samples and calculated the change in the ^13^CO_2_:^12^CO_2_ ratio brought about by the metabolism of the labeled substrate. Two standard output variables were considered here: the ^13^C breath test half-dose recovery time (^13^CT_1/2_) and the delta over baseline (DOB) values.

#### Blood sampling.

Participants were cannulated in a forearm vein by a research nurse. Nine serial blood samples were collected during the study day. The plasma was rapidly separated by centrifugation at 3000 × *g* and 4°C, snap-frozen in liquid nitrogen, and stored at −80°C for later assay. The assay methods were described previously (10). Briefly, 1 mL plasma was added to 2 mL 96% ethanol and mixed on a vortex in a glass tube. The tubes were allowed to stand on the bench for 10 min before centrifugation at 3000 *g* for 10 min. The supernatant was decanted into another glass tube and evaporated to dryness in vacuum before being redissolved in 1 mL PBS. The resulting concentrations of CCK were then measured by RIA with commercially available kits according to the manufacturer’s instructions (EURIA-CCK; Euro-Diagnostica, obtained from Immunodiagnostic Systems). The samples and the standards competed with ^125^I-CCK-8 sulfate in binding to antibodies of CCK-8 sulfate. The antibody bound with ^125^I-CCK-8 sulfate was separated, and the radioactivity of the bound fraction measured in a gamma counter.

#### Data analysis.

Gastric and the gallbladder volumes were determined as previously described (10, 23, 24) by manual segmentation of each image slice by using Analyze software (Analyze9; Biomedical Imaging Resource, Mayo Foundation). Because the individual gastric volume/time curves were linear (see Results), the gastric half-emptying time (T_50%_) was calculated from a simple linear fit to the postprandial volumes, without forcing the intercept to the volume at t = 0. The gallbladder data were normalized to each individual’s fasting volume and expressed as percentage contraction from the fasting state. The SBWC was measured by manual segmentation by using techniques previously described and validated by intubation studies (19, 20) and software written in house in IDL (Research Systems). The areas of the water and fat peaks were measured from the MRS spectra by using in-house software written in Matlab, and lipid:water ratios were calculated. The AUCs were calculated by using the trapezoidal method.

#### Power and statistical methods.

The main outcome measure of this study was the area under the gastric-emptying curve (AUC). Previous studies in 9 healthy participants (10) showed that a 15%-fat emulsion meal (3.6-μm droplet size) that was stable in the gastric environment emptied from the stomach with a mean ± SD AUC of 76 ± 24 L ⋅ min, whereas a comparable fat emulsion meal that was unstable in the gastric environment emptied from the stomach nearly 3 times faster with an AUC of 29 ± 14 L ⋅ min. Assuming similar variability, we calculated that we could detect a similar change in gastric emptying between Coarse+LBG and Coarse treatments with *P* < 0.05 and 90% power by using *n* = 5 participants in a paired design. We recruited 11 participants to increase power for the other outcomes.

Data are expressed as means ± SEMs. Tests for normality of the data were carried out by using Shapiro-Wilk test. In keeping with the hypotheses and the nature of the test meals, we separately compared Coarse vs. Coarse+LBG and Coarse+LBG vs. Fine+LBG treatments. The significance of differences was evaluated where appropriate by using 2-factor repeated-measures ANOVA, 2-factor repeated-measures ANCOVA [ANCOVA for the satiety VAS scores as recommended (25)], and 1-factor ANOVA of the AUCs followed by post hoc individual *t* tests with the use of Bonferroni correction for multiple comparison or paired *t* test. The software programs GraphPad Prism 5 (GraphPad Software), SPSS 22 (IBM UK), and SAS 9.3 (SAS Institute) were used. Differences were considered significant at *P* < 0.05.

## Results

**FIGURE 2:**
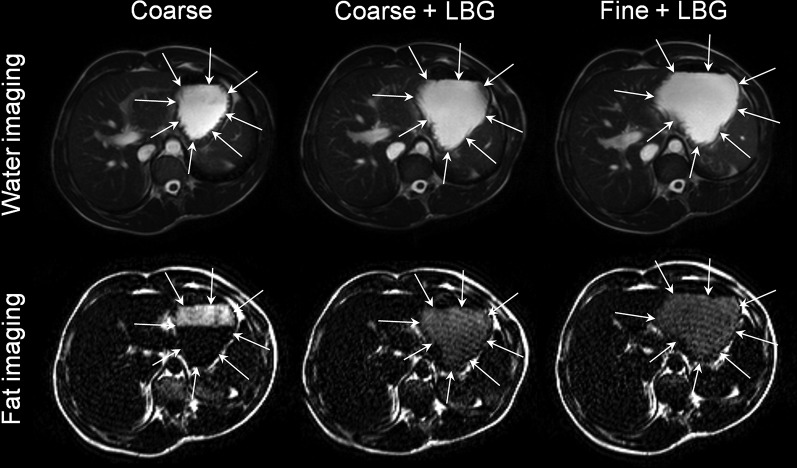
The upper part of the panel shows typical balanced turbo field echo (water) images taken across the stomach of the same healthy young adult at time t = 2 h after consumption of each of the 3 test meals containing a 20% sunflower oil in water emulsion on the 3 separate study days: Coarse, Coarse+LBG, and Fine+LBG. The lower part of the panel shows the corresponding fat-only images. These show that the LBG-stabilized emulsions did not phase separate in the stomach, whereas the Coarse meal shows a clear creamed fat layer on top of the stomach contents. Coarse, 20% oil and water emulsion with 6-μm mean droplet size; Coarse+LBG, 20% oil and water emulsion with 6-μm mean droplet size and 0.5% locust bean gum; Fine+LBG, 20% oil and water emulsion with 0.4-μm mean droplet size and 0.5% locust bean gum; LBG, locust bean gum.

The method of production of the emulsion meals was reproducible as shown by the relatively small variation in droplet sizes: the surface area mean diameter D[3,2] was 6.0 ± 0.9 μm for Coarse, 6.3 ± 0.4 μm for Coarse+LBG, and 0.44 ± 0.04 μm for Fine+LBG treatments. The addition of LBG had very little effect on droplet sizes, as desired. All young adult participants tolerated the study procedures well. Good-quality images of the fat emulsion meals in the stomach were obtained, as shown in **Figure 2**. The layering of the Coarse meal is easily seen in contrast to the appearance of the LBG-containing meals. This confirms that the addition of LBG and the consequent increase in viscosity did increase intragastric stability and reduced creaming as desired, particularly for the Fine+LBG meal, which appeared to be homogeneous throughout the stomach.

### Gastric emptying.

**FIGURE 3:**
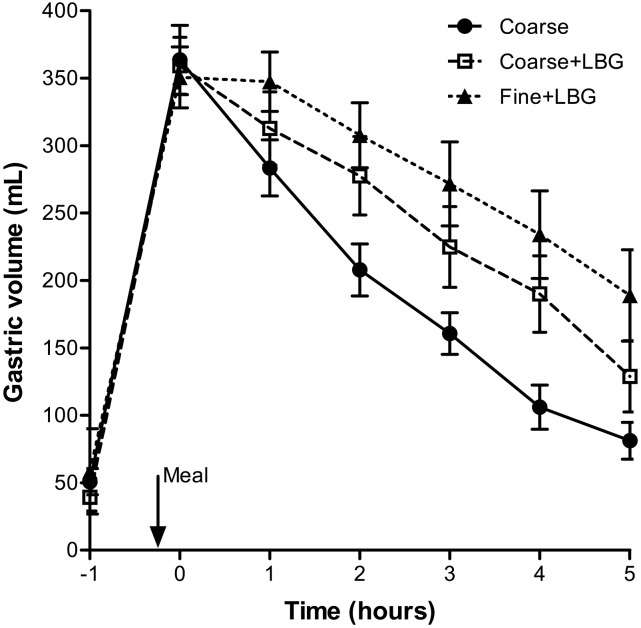
Volume of the gastric contents over time for healthy young adults after they consumed the 3 meals containing a 20% sunflower oil in water emulsion on the 3 separate study days: Coarse, Coarse+LBG, and Fine+LBG. Values are means ± SEMs, *n* = 11. The arrow indicates the meal time. Coarse, 20% oil and water emulsion with 6-μm mean droplet size; Coarse+LBG, 20% oil and water emulsion with 6-μm mean droplet size and 0.5% locust bean gum; Fine+LBG, 20% oil and water emulsion with 0.4-μm mean droplet size and 0.5% locust bean gum; LBG, locust bean gum.

The time courses of the emulsions’ mean intragastric volumes are shown in **Figure 3**. Gastric emptying was linear for all 3 emulsions (overall mean linear fit: *R*^2^ = 0.89 ± 0.02). The Fine+LBG treatment emptied from the stomach slower than the Coarse+LBG treatment, and this emptied slower than the Coarse emulsion treatment as shown by the T_50%_ values in **Table 1**. The corresponding mean AUCs are also shown in Table 1. Hence, adding LBG to the larger emulsion treatment (Coarse+LBG) decreased emptying rates compared with the Coarse treatment. Reducing droplet size further reduced gastric-emptying rates, with gastric volumes for the Fine+LBG treatment remaining higher throughout the study.

**TABLE 1:** MRI, physiologic, and behavioral variables in healthy men and women after consumption of the 3 fat emulsion meals^1^

	Fat emulsion meal		
	Coarse	Coarse+LBG	Fine+LBG
Gastric volume AUC, mL ⋅ h	1190 ± 90^b^	1450 ± 140^a^	1640 ± 143^a^
T_50%_, min	180 ± 9	230 ± 22	330 ± 61
Lipid:water ratio AUC, AU ⋅ h			
Upper part	1.90 ± 0.20^a^	0.90 ± 0.07^b^	0.90 ± 0.05^b^
Lower part	0.30 ± 0.05^b^	0.70 ± 0.06^a^	0.70 ± 0.04^a^
Percentage gallbladder contraction AUC, % ⋅ h	300 ± 21	310 ± 34	300 ± 33
SBWC AUC, mL ⋅ h	1720 ± 185^b^	2020 ± 169^a^	2270 ± 180^a^
CCK AUC,^2^ pmol/L ⋅ min	1520 ± 239^b^	2100 ± 250^a^	1990 ± 258^a^
^13^C Breath test			
DOB AUC, ‰ ⋅ min	3460 ± 324^a^	2820 ± 151^b^	3140 ± 170^a,b^
^13^CT_1/2_, min	260 ± 21^b^	390 ± 40^a^	470 ± 43^a^
VAS AUC, scores ⋅ h			
Fullness	20 ± 3	20 ± 4	20 ± 4
Hunger	30 ± 5	30 ± 4	30 ± 5
Prospective food consumption VAS AUC, scores ⋅ h	30 ± 5	30 ± 3	30 ± 4
Energy intake from ad libitum meal, kJ	4060 ± 390^a^	3680 ± 409^b^	3270 ± 368^c^

1 Values are means ± SEMs, *n* = 11 unless otherwise indicated. Labeled means in a row without a common superscript letter differ, *P* < 0.05. AU, arbitrary units; CCK, cholecystokinin; Coarse, 20% oil and water emulsion with 6-μm mean droplet size; Coarse+LBG, 20% oil and water emulsion with 6-μm mean droplet size and 0.5% locust bean gum; DOB, delta over baseline; Fine+LBG, 20% oil and water emulsion with 0.4-μm mean droplet size and 0.5% locust bean gum; SBWC, small bowel water content; T_50%_, gastric half-emptying time; VAS, visual analog scale; ^13^CT_1/2_, ^13^C breath test half-dose recovery time.

2 *n* = 10.

### Spectroscopy of the intragastric fat:water ratio.

**FIGURE 4:**
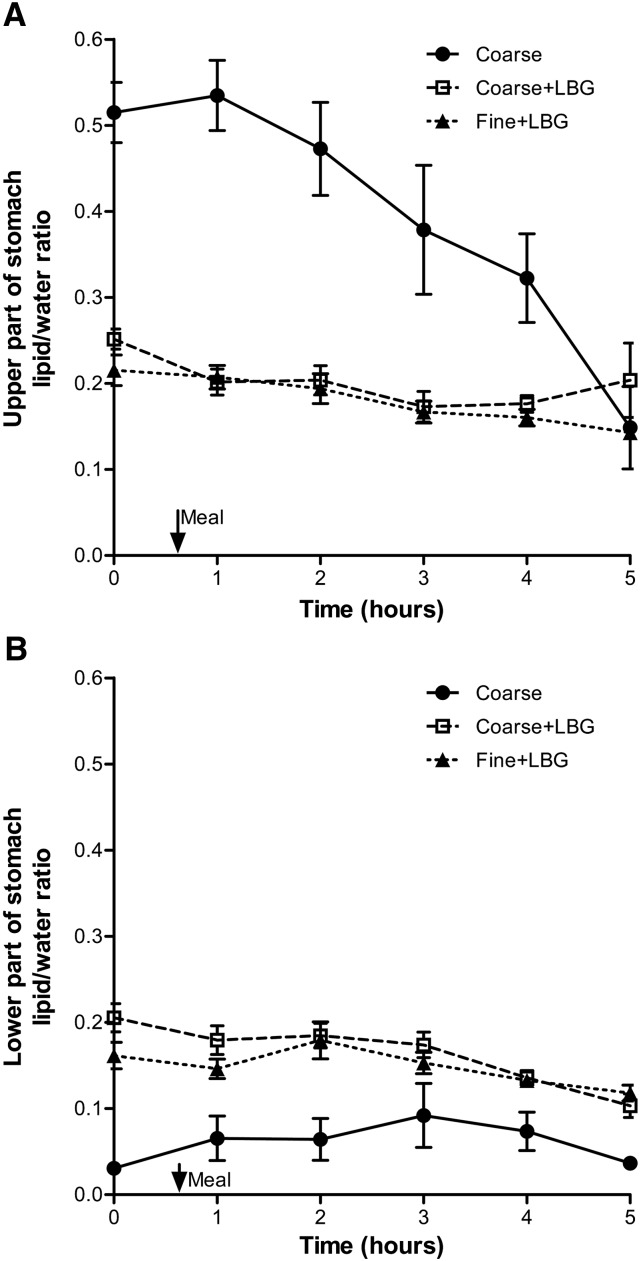
Lipid:water ratio measured from spectroscopy voxels placed in the upper (A) and the lower (B) part of the stomach of healthy young adults after consumption of the 3 meals containing a 20% sunflower oil in water emulsion on the 3 separate study days: Coarse, Coarse+LBG, and Fine+LBG. Values are means ± SEMs, *n* = 11. The arrow indicates the meal time. Coarse, 20% oil and water emulsion with 6-μm mean droplet size; Coarse+LBG, 20% oil and water emulsion with 6-μm mean droplet size and 0.5% locust bean gum; Fine+LBG, 20% oil and water emulsion with 0.4-μm mean droplet size and 0.5% locust bean gum; LBG, locust bean gum.

An example of positioning of the MRS voxel in the upper and lower part of the stomach contents is shown in **Supplemental Figure 3**. **Figure 4** shows the time courses of the lipid:water ratio calculated from the MRS data including the overall mean for the upper voxel and lower voxel. The data show significant differences between the Coarse and the Coarse+LBG treatment, with the upper layer of the Coarse treatment having a higher lipid:water ratio AUC than the lipid:water ratio AUC for the Coarse+LBG emulsion treatment (AUC, *P* < 0.001). The difference was 2.5-fold at t = 1 h. The lower layer of the Coarse treatment had a much lower lipid:water ratio AUC than the Coarse+LBG emulsion treatment (*P* < 0.0001). This is in keeping with the behavior shown in Figure 2. There was no difference between the Coarse+LBG treatment lipid:water ratio AUC and the Fine+LBG treatment lipid:water ratio AUC (upper layer, *P* = 0.75; lower layer, *P* = 0.87), confirming the desired lack of differences in the intragastric distribution between these 2 different droplet size emulsion systems containing LBG to stabilize them in the stomach.

### Gallbladder volumes.

The percentage of gallbladder contraction is shown in **Supplemental Figure 4**. There were no differences between treatments for adding LBG (*P* = 0.71) or droplet size effects (*P* = 0.79) (Table 1).

### SBWC.

**FIGURE 5:**
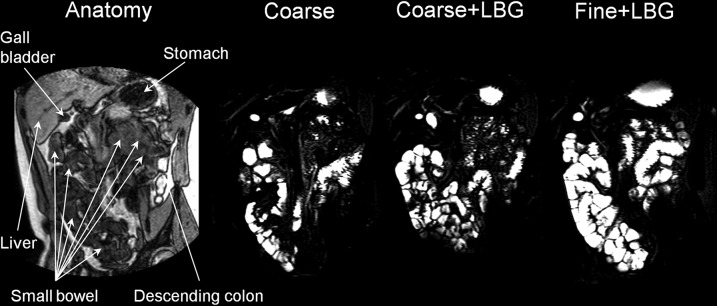
On the left side of the panel is a coronal anatomic “roadmap” dual-echo MRI image. The other images are corresponding small bowel water images taken across the small bowel of the same healthy young adult at time t = 4 h after consumption of each of the 3 meals containing a 20% sunflower oil in water emulsion on the 3 separate study days: Coarse, Coarse+LBG, and Fine+LBG. These images show the large amount of freely mobile fluid present in the small bowel in response to the fat emulsion meals. Coarse, 20% oil and water emulsion with 6-μm mean droplet size; Coarse+LBG, 20% oil and water emulsion with 6-μm mean droplet size and 0.5% locust bean gum; Fine+LBG, 20% oil and water emulsion with 0.4-μm mean droplet size and 0.5% locust bean gum; LBG, locust bean gum.

**FIGURE 6:**
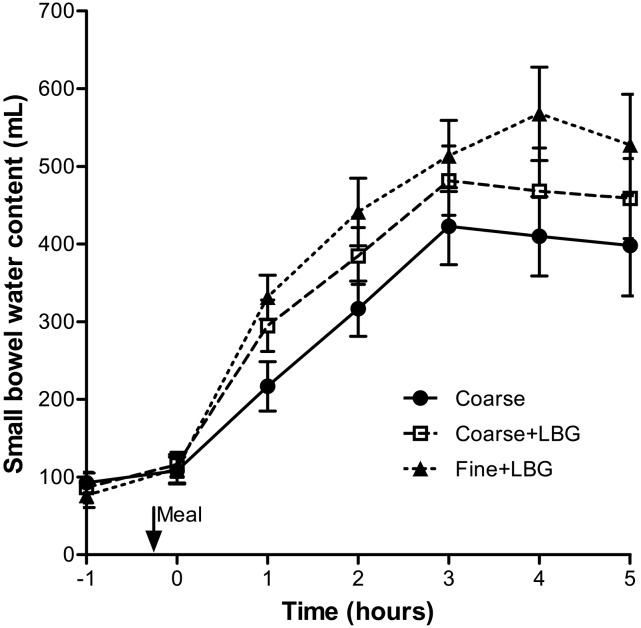
Small bowel water content volume over time for healthy young adults after they consumed the 3 meals containing a 20% sunflower oil in water emulsion on the 3 separate study days: Coarse, Coarse+LBG, and Fine+LBG. Values are means ± SEMs, *n* = 11. Coarse, 20% oil and water emulsion with 6-μm mean droplet size; Coarse+LBG, 20% oil and water emulsion with 6-μm mean droplet size and 0.5% locust bean gum; Fine+LBG, 20% oil and water emulsion with 0.4-μm mean droplet size and 0.5% locust bean gum; LBG, locust bean gum.

**Figure 5** shows sample SBWC images, and **Figure 6** shows the mean SBWC volume time courses. The addition of LBG to the Coarse emulsion significantly increased SBWC (*P* = 0.0077, Table 1) and reducing droplet size increased SBWC (*P* = 0.0086, Table 1). The mean time to the peak value in the SBWC data showed a trend to be longer for the Fine+LBG meal treatment (4 h) than for both the Coarse+LBG and the Coarse treatments (3 h).

### Plasma CCK.

**Supplemental Figure 5** shows the mean CCK plasma concentration time courses. Adding LBG to the Coarse emulsion significantly increased the AUC of plasma CCK compared with the Coarse treatment (*P* = 0.0274, Table 1). There were no differences in plasma CCK between Fine+LBG and Coarse+LBG treatments (*P* = 0.62).

### Breath test.

Adding LBG to the larger emulsion (Coarse+LBG) significantly increased the ^13^CT_1/2_ and reduced the DOB compared with the Coarse treatment. The appearance of the ^13^C-label in the breath was faster for the Coarse emulsion meal than for the LBG-stabilized meals in the first 2 h as shown in **Supplemental Figure 6**. The DOB was higher for the Fine+LBG treatment after 3 h postprandially, and the difference was modest but significant compared with the Coarse+LBG treatment (*P* < 0.0001). In contrast, the DOB was higher for the Coarse emulsion treatment over the first 3 h postprandially (*P* < 0.0001).

### Satiety and food intake.

The mean time courses of the different satiety VAS scores are shown in **Supplemental Figure 7**. There were no differences between treatments (ANCOVA *P* value range: <0.61–0.98).

**FIGURE 7:**
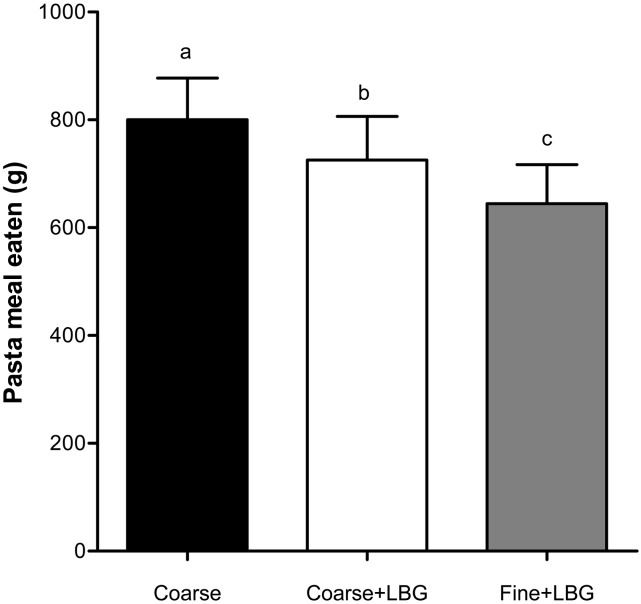
Weight of food consumed ad libitum from a pasta meal by healthy young adults after they consumed the 3 meals containing a 20% sunflower oil in water emulsion on the 3 separate study days: Coarse, Coarse+LBG, and Fine+LBG. Values are means ± SEMs, *n* = 11. Labeled means without a common letter differ, *P* < 0.05. Coarse, 20% oil and water emulsion with 6-μm mean droplet size; Coarse+LBG, 20% oil and water emulsion with 6-μm mean droplet size and 0.5% locust bean gum; Fine+LBG, 20% oil and water emulsion with 0.4-μm mean droplet size and 0.5% locust bean gum; LBG, locust bean gum.

**Figure 7** shows the effects of feeding the 3 fat emulsion meals on food intake. Adding LBG to the Coarse emulsion treatment significantly reduced the amount of pasta meal eaten afterward (Coarse+LBG compared with Coarse, *P* < 0.05). This represented a 9% reduction compared with the Coarse+LBG treatment, which is equivalent to an average of 379 kJ less energy consumed (Table 1). Reducing droplet size also significantly reduced the amount of pasta meal eaten afterward (Fine+LBG compared with Coarse+LBG, *P* < 0.05), and represented a 11% reduction compared with the Coarse+LBG emulsion treatment, which is equivalent to an average of 411 kJ less energy consumed (Table 1).

## Discussion

As we hypothesized, increasing the intragastric stability of the Coarse fat emulsion meal without altering emulsion size by adding LBG (i.e., preventing creaming) slowed gastric emptying, increased SBWC, and slowed the appearance of the ^13^C label in the breath of subjects, which is indicative of a reduced rate of absorption. These effects were followed by a significantly decreased food intake. Layering of fat (creaming) at the top of the lumen away from the pylorus would lead to delivery to the duodenum and small bowel of a fat-depleted watery phase during early gastric emptying. This would, in turn, lead to less activation of the inhibitory feedback including CCK and hence faster reduction in gastric volumes of the Coarse emulsion without LBG. This was confirmed by our finding of a higher AUC for CCK when LBG was added to the meal. The LBG-stabilized meals ensured steady delivery of fat to the duodenum throughout gastric emptying, triggering duodenal receptors to activate the feedback loop to slow gastric emptying. The gastric behavior and gastric-emptying response to these fat emulsions were analogous to those previously observed with the use of acid-stable and acid-unstable fat emulsions (10). Intubation studies showed that intragastric layering of small amounts of finely emulsified fat (4 g) delayed lipid absorption and increased plasma CCK, with differences being mostly modest (26).

The presence of LBG prevented layering in the stomach, thus allowing inferences on the effect of droplet size to be drawn in support of our second hypothesis, although some differences such as viscosity and some residual gastric creaming remained and need to be taken into account when interpreting the results. Decreasing fat droplet size in an LBG-stabilized fat emulsion slowed gastric emptying, increased SBWC, and reduced food intake. The Fine+LBG emulsion showed a trend to empty more slowly than the matched Coarse+LBG emulsion (although T_50%_ differences were not significant, *P* < 0.07), possibly reflecting increased activation of duodenal fat receptors by the more finely emulsified droplets, which therefore present a larger surface area for hydrolysis. Although CCK release was increased by adding LBG to the meal, no further increase was observed with reduction in particle size, possibly because maximal release had already been achieved. Earlier invasive intubation studies showed that instilling finer intragastric emulsions led to initially more rapid fat hydrolysis, which showed that reducing emulsion size speeds digestion, which would be predicted to enhance release of gastrointestinal peptides and hence slow gastric emptying (14). Previous work showed that duodenal delivery of small amounts (6 g) of fine fat droplets (∼1 μm) within the context of a meal replacer reduced hunger more than did coarse fat droplets, although without concomitant changes in CCK or PYY, possibly due to the small dose (17).

We observed a striking postprandial increase in SBWC, which has not been previously observed with high-carbohydrate rice meals in which SBWC decreases and then stabilizes at ∼200 mL (20). The continuing increase in SBWC after our high-fat meals to a peak at 4 h of 568 mL (range: 150–854 mL) for the Fine+LBG emulsion was also very different from the decrease in SBWC seen after bread-containing meals in which the mean SBWC was 55 mL (range: 11–153 mL) (27). We hypothesize that this reflects pancreatic and biliary secretions, which would be predicted to be much greater with these high-fat meals. This effect was significantly increased by LBG and further increased by reducing droplet size. although this difference was not significant (*P* < 0.06).

Initial gallbladder contraction differences, which could be expected given the different intragastric behavior of the meals (10), may have been missed because of the hourly sampling frequency. The delayed ^13^C breath peak reflects the delayed gastric emptying and hence differences in availability of the fat for digestion and metabolism.

The significant changes observed in plasma CCK were not reflected in the VAS scores in this study, possibly due to the high variability in the scores; the study was not powered to detect changes in satiety VAS. However, significant changes were measured in objective food intake.

The addition of LBG to the Coarse emulsion reduced food intake by ∼9%. Reducing droplet size in a gastric-stabilized emulsion reduced food intake by another 11% (Coarse+LBG vs. Fine+LBG). The total reduction in food intake was therefore 20% or 790 kJ, which can be considered substantial in light of long-term accumulation of relatively small daily “energy gaps” in obesity (28). Hill et al. (28) described that by reducing caloric intake by 226 kJ (50 kcal)/d, weight gain could be prevented in 90% of the population. This suggests that the mechanism of reduction in food intake we observed is meaningful when assessing food manipulations aimed at preventing obesity and overweight.

When designing the study we expected that a Coarse fat emulsion would cream in the stomach more than a Fine emulsion, introducing a confounding variable in the output from the stomach itself—hence, confounding the assessment of the gastrointestinal and satiety responses to different droplet sizes. To control for this problem, we integrated a small amount of food thickener in order to stabilize Coarse and Fine emulsion meals against creaming inside the stomach. LBG was chosen for this because it is relatively inert, quite resistant to the acidic environment of the stomach, and does not disturb the droplet size profiles too much. The fat droplet size delivered from the stomach to the duodenum may change in the stomach from the initial, ingested size due to competing processes of coalescence (14) and gastric emulsification (29), but this would be reduced by the presence of LBG.

One limitation of this work is the physiologic variability of individual human healthy subjects. The studies were powered appropriately for the main outcome; however, secondary outcomes would have benefited from larger numbers of subjects due to variability. Another limitation is the horizontal imaging position, which is necessary due to the scanner configuration. In the supine position there is a risk that floating creamed fat layers may enter the duodenum first, preventing the effect on gastric emptying, which is something we tried to avoid by imaging the subjects while in a tilted position to keep the left side uppermost. When effects of layering are prevented, the effects of body position on gastric emptying are small (30) and posture effects on satiety (31) should be minimized. The zero-shear viscosity of the LBG-stabilized fat emulsions was 3 orders of magnitude higher than that for the Coarse treatment. Consequently, effects of viscosity on gastric behavior cannot be ruled out, although the effects of viscosity of LBG solutions on gastric emptying are expected to be modest (32). Shear thinning of these emulsions in vivo would also reduce the differences. Possible effects of differences in perceived viscosity, particularly between the sample without LBG and the 2 samples with LBG, would have been minimized by randomization and blinding.

In summary, MRS and MRI can be used to monitor the gastrointestinal response to different fatty emulsions serially and noninvasively. A highly emulsified intragastrically stable emulsion led to delayed gastric emptying, increased SBWC, and reduced consumption of food at the end of the study day. Manipulating food microstructure, especially intragastric stability and fat emulsion droplet size, can influence human gastrointestinal physiology and food intake. This could show advantages in products designed for infant feeding, weight management, or clinical nutrition.

## Footnotes

10 Abbreviations used: CCK, cholecystokinin; Coarse, 20% oil and water emulsion with 6-μm mean droplet size; Coarse+LBG, 20% oil and water emulsion with 6-μm mean droplet size and 0.5% locust bean gum; DOB, delta over baseline; Fine+LBG, 20% oil and water emulsion with 0.4-μm mean droplet size and 0.5% locust bean gum; LBG, locust bean gum; MRS, magnetic resonance spectroscopy; PP, pancreatic polypeptide; PYY, peptide YY; SBWC, small bowel water content; TE, echo time; TR, repetition time; T_50%_, gastric half-emptying time; VAS, visual analog scale.
